# SimplyFire: An Open-Source, Customizable Software Application for the Analysis of Synaptic Events

**DOI:** 10.1523/ENEURO.0326-23.2023

**Published:** 2024-01-19

**Authors:** Megumi Mori, Andrew Rosko, Jill Farnsworth, Guadalupe Carrasco, Parisa Broomandkhoshbacht, Kristeen Pareja-Navarro, A. Pejmun Haghighi

**Affiliations:** ^1^The Buck Institute for Research on Aging, Novato 94947, California; ^2^Gladstone Institute of Neurological Disease, Gladstone Institutes, San Francisco 94158, California

**Keywords:** data analysis, electrophysiology, open-source software, software package, synaptic events

## Abstract

We have developed an open-source software for neuroscientists to analyze electrophysiological recordings. Named SimplyFire, the software gives the users the flexibility to analyze a variety of recordings using an interactive graphical user interface or as an importable Python package. The software features a simple plugin structure that allows users to create and deploy various electrophysiology analysis tools. SimplyFire is pre-packaged with tools commonly used in electrophysiology, such as noise filtering, trace averaging, miniature analysis, and trace exporting. We discuss in detail the algorithm behind the different features of the analysis tool. We verify the accuracy of the algorithm by testing the software using computer-generated traces with known true values of the events. SimplyFire will be distributed under the GPLv3.0 license. The open nature of this software will allow interested investigators to modify and expand the software for additional capabilities as needed. We believe this software will not only compete with commercially available software packages but will also present a powerful tool to meet the current and unmet needs of electrophysiologists.

## Significance Statement

Accurate and efficient analysis of synaptic events is a challenge and requirement for many electrophysiologists. Several proprietary programs have been developed commercially over the years that offer options for analyzing synaptic events, but the algorithms behind such programs are often hidden, and they rarely offer any flexibility for the generation of user-based modules aimed at specific requirements of individual users. Our newly generated software, SimplyFire, meets this unmet need by offering a powerful and efficient algorithm for analyzing synaptic events in Python, while allowing flexibility to adapt to specific users’ needs. Importantly, SimplyFire is freely available to the community and can evolve and be expanded by users.

## Introduction

Detection and analysis of synaptic events, such as evoked and spontaneous/miniature excitatory postsynaptic currents (EPSCs and mEPSCs) or inhibitory postsynaptic currents [induced pluripotent stem cells (iPSCs) and mIPSCs], are essential for understanding changes in synaptic plasticity and circuit function. In particular, because of their small size, fast kinetics, and stochastic nature, mEPSCs and mIPSCs require specialized computational methods for fast and efficient analysis. Several proprietary programs have been developed commercially over the years to measure these synaptic events, but the algorithms behind such programs are often hidden, and they rarely offer any flexibility for the generation of user-based modules aimed at the specific requirements of individual users. Recently, efforts have been made to develop free, open-source algorithms for electrophysiology analysis (Siegle et al., 2017; Nasiotis et al., 2019; Kim et al., 2021); however, efficient and user-friendly open-source programs remain sparse. To address this issue, we have developed SimplyFire, an open-source graphical user interface (GUI) package for detecting and analyzing both spontaneous miniature synaptic events as well as evoked synaptic currents and potentials.

We have chosen to develop SimplyFire in Python, a free, open-source programming language. Python is a simple high-level programming language that is popular among biologists, making it more likely that the software can be read and understood in its entirety by many electrophysiologists. Moreover, Python does not require compilation, allowing for fast distribution and development.

SimplyFire is pre-packaged with other post-acquisition tools, such as noise reduction, baseline subtraction, and statistical analyses. SimplyFire can thus be incorporated into the analysis workflow immediately after the acquisition of the electrophysiology data. Additionally, the software is equipped with the capacity to incorporate packages developed by users, providing an opportunity for the software to grow with community input. Here, we describe the architecture of the algorithm underlying SimplyFire. We further assess the performance of SimplyFire to detect synaptic events, using both recorded and simulated data, as compared to a popular commercial software that is no longer supported. Finally, we demonstrate the ability of SimplyFire to single-handedly complete the post-acquisition workflow using recordings of *D. melanogaster* larval neuromuscular junction (NMJ).

## Methods

### Artificial data generation

For the data analyzed in Figure 3*A–E* and the sine wave noise (data not shown), the artificial miniature postsynaptic events were generated according to the equation as follows:

$$Y=A\left(1-e^{-\frac{t}{\tau_{1}}}\right)*\;e^{-t/\tau_{2}},$$

where the time constants were uniform random variables with ranges $\;\;2<\tau_{1}<10\;\;2<\tau_{2}<15$ and the scaling factor A was chosen independently for each event after the fact such that the amplitude (the value of Y at the local maximum of the curve) was a uniformly distributed random variable between 0.5 and 2.5. This was necessary because the maximum value of the function is dependent not merely on A, but on the relative values of the time constants as well.

Here, t is the time in milliseconds from the start of the event, the parameters were chosen such that the time of the local maximum ranged from approximately 2–10 ms, and the time after the maximum for the curve to decay to 1/e of the maximum value ranged from approximately 5–20 ms. For comparison with SimplyFire output, the rise time from 10 to 90% of the maximum and the decay constant were calculated according to the actual curve, not from $\tau_{1}$ and $\tau_{2}$, respectively, as in actuality both time constants influence both of these parameters through changing the peak position.

The equation of each event was evaluated out to $t_{\max}=5*(\tau_{1}+\tau_{2})$ to ensure that the amplitude decayed to baseline before the next segment of baseline was added to the data set as a spacer between this event and the subsequent event. The length of this spacing between successive events (i.e., between $t=t_{\max}$ of one event and t=0 of the next event) was chosen as a uniformly distributed integer random variable between 100 and 500 data points (10 and 50 ms).

Sharper events more closely approximating current data (Figs. 3*F*, 4) were generated by a piecewise function given by the following:

$$y(t)=\frac{At}{t_{\mathrm{rise}}}\;,t\leq t_{\mathrm{rise}}$$

$$y(t)=Ae^{-(t-t_{\mathrm{rise}})/\tau_{\mathrm{decay}}}\;,t>t_{\mathrm{rise}}$$

Here, the full rise time, amplitude, and decay constant are directly given by the formulas (*t*_rise_, *A*, and $\tau_{\mathrm{decay}}$, respectively).

For the sine wave noise, a function Y=0.02sin2πt was added to the idealized recording to produce the mock recording to be analyzed with SimplyFire. Thus, one full period of the sine wave was 1 ms. For the random noise (Fig. 3), a series of independent Gaussian random noise variables $n_{i}$ was computed with mean 0 and standard deviation $0.02/\sqrt{5}$. Then, the individual samples $y_{i}$ of the mock recording were calculated from the corresponding idealized values $y_{i\mathrm{ideal}}$ according to the following:

$$y_{i}=y_{i,\mathrm{ideal}}+\overset{i}{\underset{\;j=i-5}{\sum }}\;n_{j}$$

This produces a time series where the noise offset of each data point is partly correlated with the offsets of the preceding and subsequent data over a window size of eleven data points, better mimicking the drift characteristics of real recording equipment than an independent deviation applied to each data point.

For the data with “hum” (Fig. 5), the random noise was generated according to the same function as above, but a hum of the form $y(t)=0.2\cdot \sin\;\left(\frac{2\pi t}{50ms}\right)$ was added in addition.

### Electrophysiology

#### Drosophila larval NMJ

We used a standard two-electrode voltage clamp as previously performed (Tsurudome et al., 2010). Briefly, wandering third instar larvae were dissected and rinsed multiple times in a cold calcium-free HL3 solution (Stewart et al., 1994). Synaptic events (mEJC and EJC) were then measured at muscle 6 in segment A3 using an amplifier (AxoClamp 2B; MDS Analytical Technologies) in two-electrode voltage clamp mode for recording miniature or evoked excitatory junctional currents (mEJCs or EJCs) or in current clamp mode to record miniature junctional potentials (mEJPs). For evoked responses, a third suction/stimulating electrode was used to stimulate the appropriate nerve bundle. All recordings were performed at room temperature in HL3 with 0.5 mM external Ca^2+^, and muscles were held at −80 mV.

#### Human iPSC-derived human neurons

Miniature excitatory postsynaptic current (mEPSC) recordings were performed on 8.5 weeks old human excitatory glutamatergic neurons that were differentiated from iPSCs using neurogenin 2 (NGN2). Whole-cell voltage clamp was performed in an external bath solution (140 mM NaCl, 5 mM KCl, 2.5 mM CaCl_2_, 2 mM MgCl_2_, 10 mM HEPES, 10 mM glucose, pH 7.4) with 0.5 mM tetrodotoxin, and the internal solution used contained 120 mM CsCl_2_, 5 mM NaCl, 1 mM MgCl_2_, 10 mM HEPES, 1 mM EGTA, 4 mM Mg-ATP, 0.3 mM Na_3_GTP, and 10 mM QX-314. The membrane potential was held at −60 mV.

### Code accessibility

All source code for SimplyFire is available on GitHub at http://github.com/HaghighiLabBuck/SimplyFire. It is released under the GNU General Public License (GPL) Version 3.

## Results

### Electrophysiology analysis workflow overview

**Figure 1.**
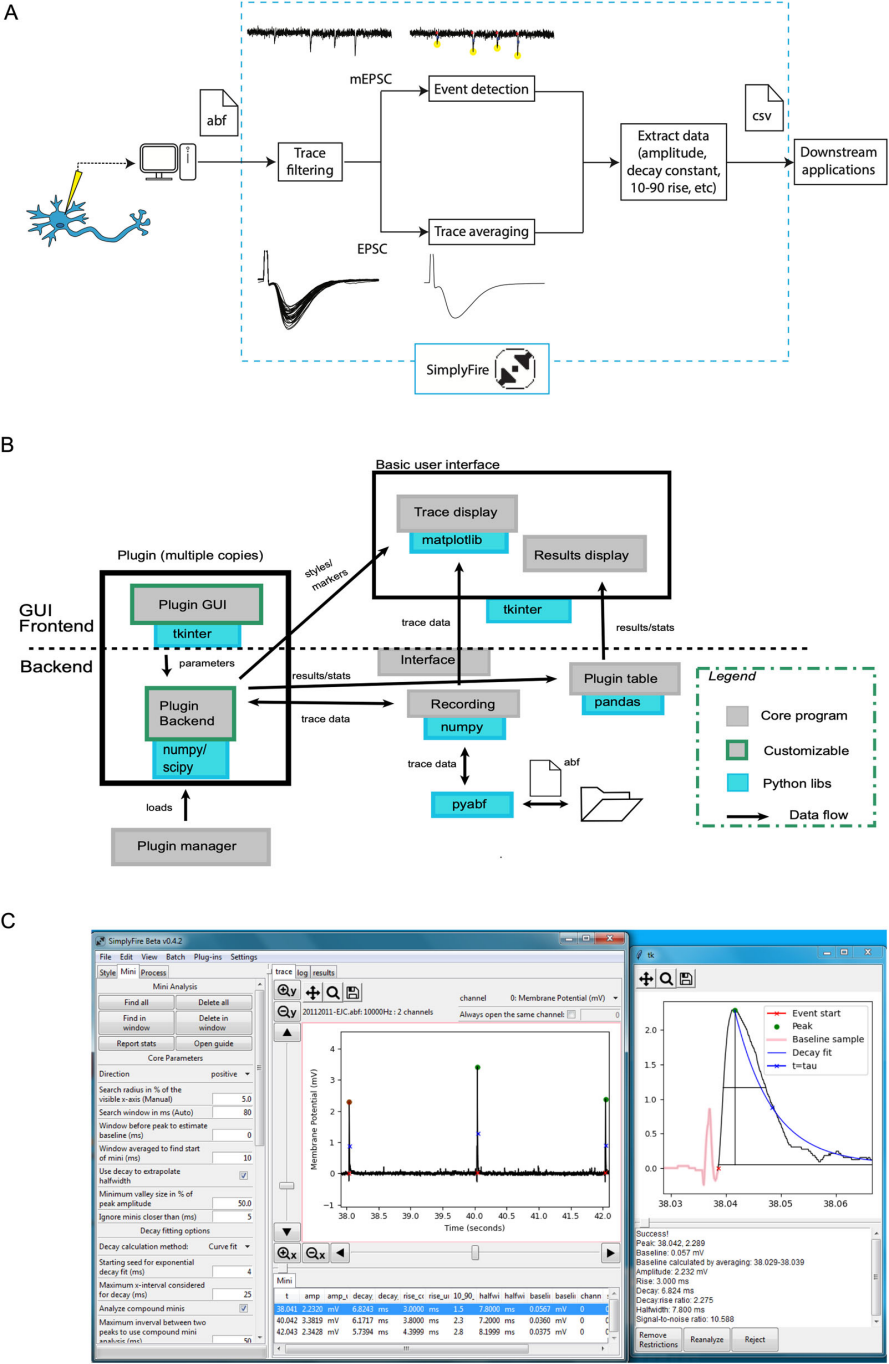
***A***, Electrophysiology analysis workflow. Recordings are captured and saved as ABF (.abf) files, which can contain unstimulated spontaneous events and/or evoked events. Both are filtered as necessary to remove noise. Then, files for spontaneous events are passed through the miniature event detection algorithm, while those for evoked events are averaged to obtain a representative response. Finally, numerical data and plot snapshots are output as .csv and image files, respectively, for publication or further analysis. Depending on user preference, it is also possible to use the “Mini” algorithm with a higher amplitude threshold to process evoked events. ***B***, Software architecture for SimplyFire. The program contains both a frontend GUI component and the backend processing component and allows for the extension of both of these components with custom plugins. Some parts of the code, such as the interpreter, that are not involved in data flow are omitted for clarity. ***C***, The user interface of SimplyFire. The main window is displayed on the left, showing the location of the trace plot, the results table, and the window displayed by the currently active plugin. The right panel shows the popup window that allows inspection of the detected parameters of a single event.

Various sets of tools are supplied in the base distribution of SimplyFire to fit the needs of electrophysiologists. A user can import a recording encoded in Axon Binary Format (ABF) into SimplyFire for analysis. Filtering, baseline subtraction, and segmentation can be applied as necessary to process the recording before analysis. Individual spontaneous and/or evoked synaptic events (hereafter called “events”) can be detected and analyzed using the “Mini” feature. Alternatively, for experiments designed to have events aligned in time (such as evoked EPSC/IPSC recordings), the traces can be averaged to obtain a representative event trace and further analyzed (Fig. 1*A*). Then, the results and statistics can be saved as a CSV file for further processing (e.g., synaptic strength quantification and significance testing).

### Software architecture

#### A modular software architecture allows customization and extensibility

The SimplyFire code is broadly organized into four portions (Fig. 1*B*):

1. The basic user interface, consisting of a main window containing space for plugin sub-windows, a plotting canvas for voltage or current time traces, an output table for results of analyses, and an action log
2. A “controller” component that handles *Tkinter* events triggered by user interaction with the app as a whole
3. A class called *recording* that acts as a wrapper around numerical arrays of time and current/voltage values, as well as invoking necessary file parsers for loading and saving of data
4. A set of plugins for processing tasks

#### The basic user interface

The GUI (Fig. 1*C*) is built using the *Tkinter* Python interface to the widget toolkit *Tcl/Tk*. The plot display panel wraps an instance of the *matplotlib* plotting library (Hunter, 2007), with custom widgets and code for navigation (zooming and panning). A toolbar at the top of the window allows load/save operations as well as opening plugin sub-windows. The log window shows a text-based list of recent actions, while the results table is designed for numerical data organized in rows, e.g., a list of detected events and their associated kinetic parameters.

In order to enable the opening of long (1 h or greater of total length among all sweeps) recordings even on computers with limited memory, care was taken to reduce the amount of data held by the plotting window to what is currently displayed within the axis bounds. User navigation triggers fetching of the required subset of current or voltage data from the *recording* object in concert with the rescaling of the axes themselves, and the *X* (time) values are generated on the fly by the *recording* object (see below).

#### The controller component

This component consists of an *interface* and an *interpreter*. The interface mediates communication between the recording object and the trace, handles file and channel selection, and implements a stack of function calls to enable undo functionality, to which plugins can push functions of their own. The interpreter handles keyboard and mouse inputs that manipulate the plot.

#### The *recording* class

This class uses the *pyabf* (Harden, 2022) Python parser for ABF files for reading and writing multi-trace recordings and also allows input and output of single data channels as CSV files. The current/voltage values are stored in NumPy (Harris et al., 2020) arrays within this class, while the time values are generated on demand when data within a given time interval is requested. Other components, particularly the plugins, can access the data for processing by querying member functions to extract a specific subset of channels and/or sweeps and a relevant interval of time.

#### The plugin module

This consists of the plugins themselves, as well as a framework of support components within the base software. All analyses are performed by individual plugins, which are loaded by a plugin manager component. A custom-made plugin can be added to the software by creating a meta-file and a Python code within the plugin module. Each plugin can have its own GUI component to request user input in the Plugin Window, listen to user interaction with the electrophysiology trace plot and data table via the *interpreter*, modify the trace plot to display additional curves or markers on top of the data traces, or add numerical entries to a *plugin table* by passing a *pandas* DataFrame (McKinney, 2010). There are several additional Python classes provided for plugins to use, such as forms and popup windows.

#### Plugins

SimplyFire includes many plugins, primarily focused on data filtering, event detection, and trace averaging, as well as for the selection of traces, navigation through the duration of a recording, and changing the stylistic attributes of the trace display.

The “Process” plugin allows several common preprocessing actions for recordings, in particular baseline subtraction, trace averaging, and filtering. The boxcar and Bessel filters are the two filtering algorithms included at this time and are based on the NumPy convolution and SciPy (Virtanen et al., 2020) signal processing functions, respectively. The trace averaging, which is intended primarily for analysis of evoked postsynaptic events, makes no attempt to align the traces in time, so it is only useful when they are pre-aligned.

The “Evoked” plugin provides basic processing operations that are useful for quantifying evoked postsynaptic events. These are calculating the minimum and maximum data point in each trace/sweep, and reporting the mean and standard deviation of these minimum and maximum values in the results table.

The “Mini” plugin is the most complex component of SimplyFire, and its algorithm is described in detail in the following section. This algorithm is used for the detection of events by filtering according to criteria, including amplitude and rise/decay times. While intended for analysis of miniature excitatory postsynaptic/junctional potentials (mEPSPs/mEJPs) or currents (mEPSCs/mEJPs), it can be in principle used for detection of any event that involves a transient de- or hyperpolarization, whenever the time of the event is unknown and noise robustness is necessary.

### Algorithm for analysis of miniature events

**Figure 2.**
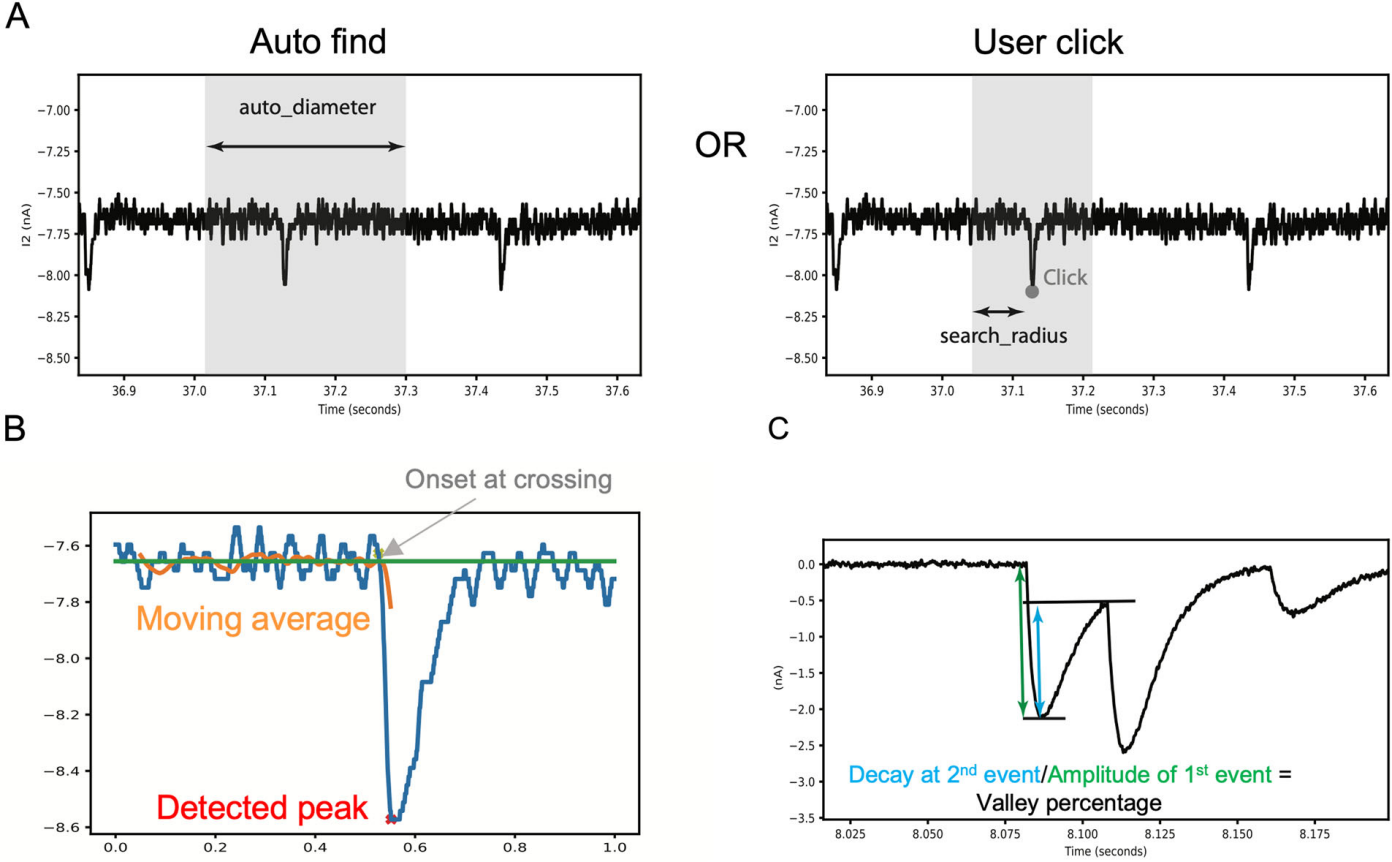
The algorithm for detection of miniature postsynaptic currents or potentials. ***A***, Depending on the usage mode, the algorithm either scans the entire recording in a series of windows of width “auto_diameter” or else searches within “search_radius” of the *X* coordinate of a mouse click, to find a global extremum. ***B***, It then proceeds leftward to detect the onset of the event using a moving average. ***C***, When enabled, compound events are detected by searching between two local extrema to find a point significantly nearer to the baseline.

The Mini Detection package in SimplyFire is used to analyze spontaneous miniature synaptic events. This plugin is used to automatically detect miniature postsynaptic events and accepts a variety of parameters from the user. The user can choose to perform two types of analyses: automated and manual. In automated analysis, the plugin scans a user-defined time window and annotates all minis found according to the parameters (Fig. 2*A*). The search radius per iteration is determined by the *auto_diameter* parameter (“Search window in ms (Auto)” within the GUI), and the algorithm shifts the window by half the diameter every iteration. In the manual mode, the plugin searches for a single mini centered around a location where the user clicks on the electrophysiology trace. The search window in the manual mode is defined by the percentage of the visible time axis, which is used to calculate the *search_radius*.

The algorithm begins by detecting the global extrema (maxima for events that lie above the baseline or minima for events that lie below) of the trace within the search window. Once a global extremum is found, the algorithm scans backward in time from this data point to locate the onset. The backward scan involves comparing each point in the trace with a moving average of a specified number of preceding data points (Fig. 2*B*). The first visited (i.e., latest in time) point that is on the opposite side of the average from the peak (i.e., below the average for events above the baseline, or vice versa) is selected as the time point immediately preceding the event, and hence the subsequent data point is the first assigned as belonging to the event.

There is a user-controlled option to use the average of a window located a fixed distance to the left of the candidate peak as the baseline instead of the moving average. This is intended to be used when the baseline immediately preceding the onset of a mini is corrupted by high noise or some other source of error. It is important to average over enough preceding data points so that local drift in the baseline or jaggedness in the rising phase of an event due to noise is not mislabeled as the time of onset.

This algorithm is loosely based on Brakel's algorithm (Brakel, 2014), with the key difference that the latter calculates an average preceding every point in the data, whereas ours only calculates one series of moving averages in the vicinity of the global maximum or minimum of each search window. This reduces the computation time considerably given the long baseline segments that must be averaged to compensate for the level of noise in our data. In addition, rather than using the standard deviation of the baseline as the cutoff to determine the transition between baseline and event, we use the simple crossing point of the smoothed and actual data. Hence, Brakel's method is best suited for detecting short sequences of outliers within data of otherwise relatively low variability, whereas ours is more efficient for detecting features defined by prolonged deviations from a varying baseline.

To determine the decay properties of the mini, the algorithm uses the data points forward from the peak. The forward curve fitting is performed using the Levenberg–Marquardt gradient descent algorithm as implemented in the *curve_fit* function of SciPy. Currently, a single exponential is the only curve that can be fit, as this makes defining a single decay constant (*τ*) for the mini meaningful and is also the most common choice of function used to model the shape of synaptic events (Kandel et al., 2000). However, for users with programming skills, other functional forms can be added by modifying the plugin code to define additional curves and passing them to the curve fitting function instead. There is also an option to define tau in a curve shape–agnostic manner as the time interval from the peak to the earliest point at which the amplitude has decayed to 1/e times the amplitude of the peak—this is equivalent to the result of the curve fitting for data that follow a true single exponential decay.

When designing the algorithm for this plugin, care was taken to ensure proper handling of both single events and “compound” events, the latter being defined as events whose distance along the time axis is too short to separate the decay of one event from the rise of another event. There is a trade-off involved here, as the second (or third and subsequent) event in a compound series is often not preceded by a segment of nearly constant (i.e., “flat”) signal that can be used to define a baseline, requiring the detection of the event onset to be based essentially on local properties of the trace immediately preceding the event. Conversely, it is important that whenever possible, the fit of an event is minimally biased by noise immediately preceding the onset. The determination of whether a pair of events constitutes a “compound” event is done by a user-provided value that defines the minimum required separation between two peaks to use single event analysis.

When this compound event detection is enabled, and when two peaks are detected that are closer in time than the specified threshold, the following algorithm is used (Fig. 2*C*). The software determines if there exists a data point between the peaks that is closer to the baseline than a user-selected percentage of the earlier peak's amplitude, which we term the “valley percentage.” If such a point is found, the later peak is considered a separate event, the point of minimum amplitude is marked as the start of the later mini, and the fitted decay curve (see above) for the earlier mini is used to subtract that mini from the region of overlap to provide a more accurate baseline (and hence amplitude) for the later mini.

There are a variety of filtering criteria that can be applied to reject unwanted events. Both minimum and maximum values for the amplitude can be specified, as for the rise time, half-width, and decay time constant. As soon as one of these conditions is found to be violated, the mini is rejected and the software proceeds to the next peak.

As various parameters can alter whether a set of data points is considered noise or a proper event, we have provided an additional feature called the *parameter guide* (Fig. 1*C*, right). This feature provides a popup window that shows the peak location, amplitude, rise time, and fitted decay curve superimposed on a zoomed-in view of the trace whenever either a manual mini detection is attempted or a pre-detected mini is selected in the results table. The guide can provide insight into why a feature was accepted or rejected as a mini or allow troubleshooting/verification when the value(s) of any detected parameter(s) seems unreasonable to the user.

### Validation of the algorithm

To determine the effectiveness of the algorithm packaged within the MiniAnalysis plugin, we generated mock recording data whose miniature event properties are known and compared the true values against the estimates generated by SimplyFire (see Methods). In order to test the resilience of the algorithm to the degree of noise that is inherent to electrophysiological recordings, we added an artificially generated noise term to these idealized data traces.

First, we modeled “noise” using a sine wave, which allowed us to precisely bind the displacement of the key features of each mini on both the *x* (time) and *y* (potential/current) axes. We found that using this noise the algorithm detected the correct time-axis position of the minis, and the estimated amplitudes matched the true values with high accuracy (*r*^2^ = 1.0 and 0.999, respectively). The estimated values for 10–90 rise time and the decay time constant (*τ*) also had a high correlation with the true values (*r*^2^ = 0.987 and 0.992, respectively).

**Figure 3.**
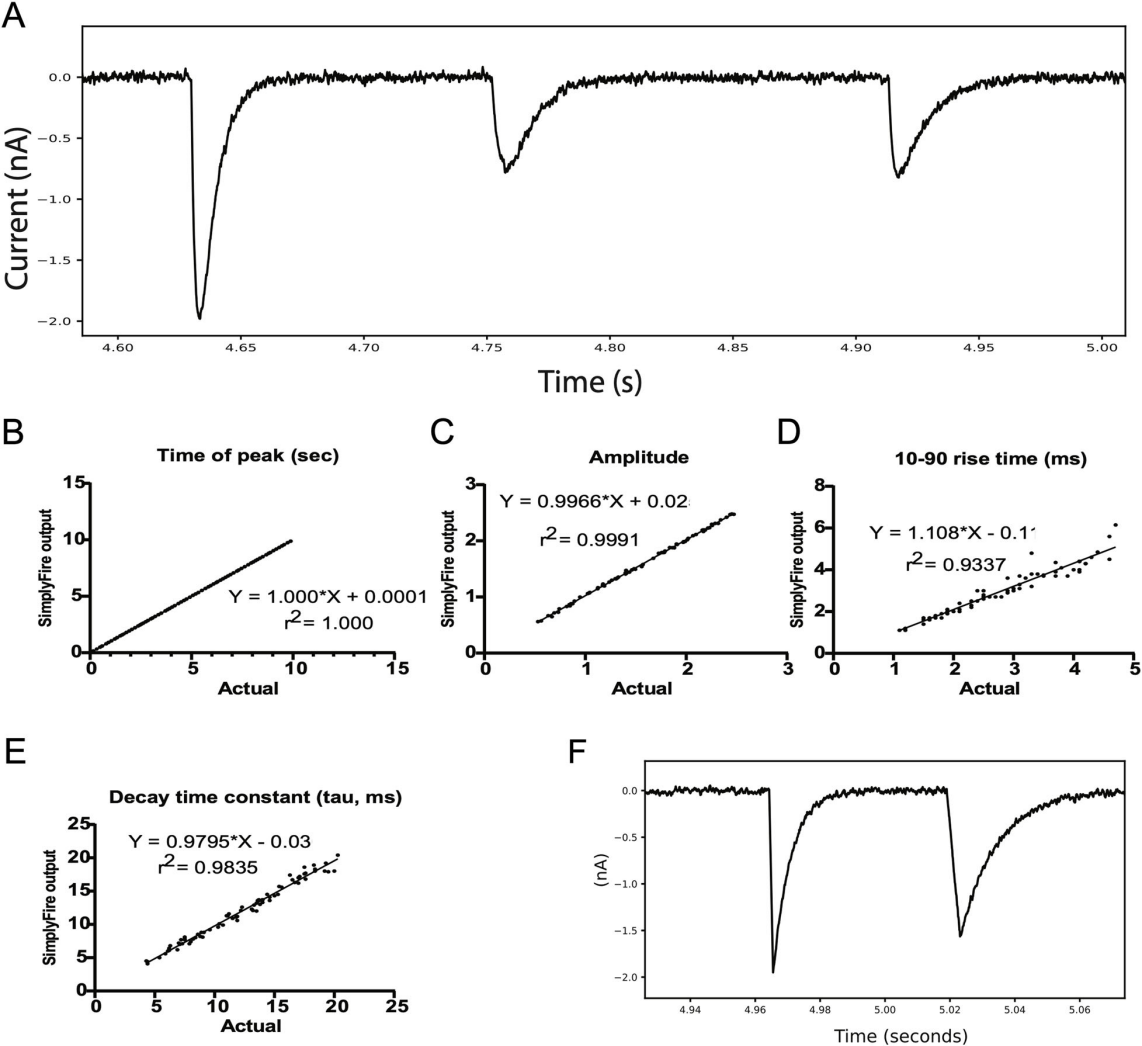
Assessment of accuracy and precision of SimplyFire on mock data. ***A***, Examples of mock miniatures generated by a double exponential, with random baseline noise. ***B–E***, Scatter plots of the time of event onset, the event amplitude, rise time from 10 to 90% of amplitude, and decay time constant as reported by SimplyFire, compared to the true values of the same parameters calculated as the data was generated. ***F***, Mock miniatures with a linear rise and single exponential decay.

To further approximate realistic recordings, we next used a random noise term that was correlated over consecutive data points, to most closely model the type of noise that is present in typical electrophysiology recordings (see Methods). The positions of the peaks in the time axis were detected accurately by SimplyFire. The amplitude estimates performed slightly worse but still very acceptably. The 10–90 rise time and the decay time constant estimates suffered slightly from the introduction of random noise, but the linear correlation between the true values and the estimates was maintained (Fig. 3*A–E*).

The mock events used in these last two data sets had the form of a product of a rising and a decaying exponential, giving the curve a rounded shape near the point of maximum amplitude, similar to membrane potential data in real recordings. We also tested sharper events consisting of a linear rise followed by a single exponential decay (Fig. 3*F*), which more closely approximates current traces in voltage clamp experiments. On this type of data, SimplyFire was better able to recover the 10–90 rise time of events (*r*^2^ = 0.992), but surprisingly was no better able to measure the decay constants (*r*^2^ = 0.981), despite them having a true single exponential form.

**Figure 4.**
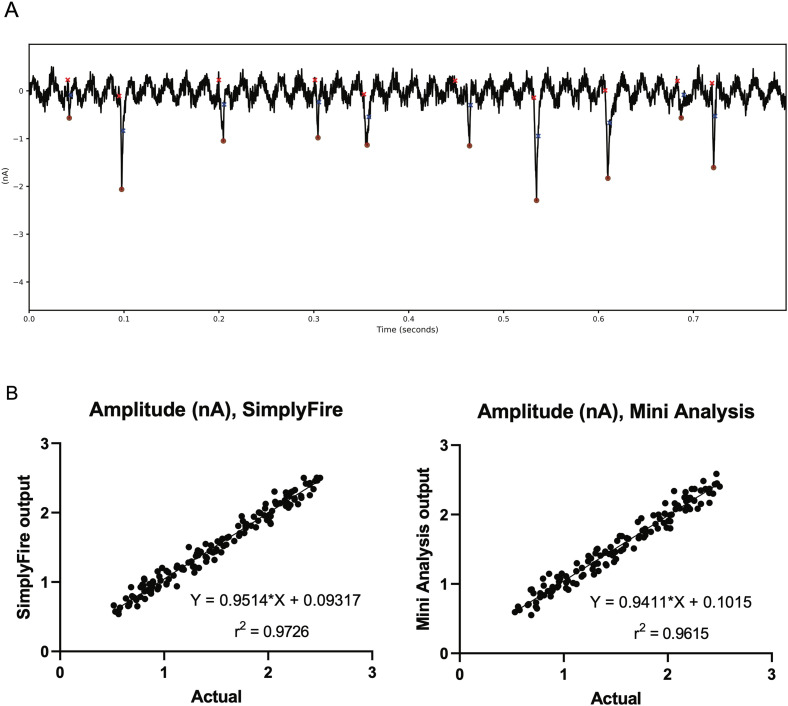
Testing of SimplyFire on mock data with a sinusoidal hum. The amplitude of the sinusoid is 0.2 nA, the amplitude of the random fluctuations is 0.1 nA, and the event amplitudes range from 0.5 to 2.5 nA. ***A***, A short segment of the mock data trace. Event peaks are detected (filled red circles), wherever they occur in the phase of the hum, but extreme points of the hum amplitude are ignored. ***B***, Correlation of detected amplitude, according to our software, with the true values. ***C***, Same as in (***B***), but with amplitudes calculated using MiniAnalysis (Synaptosoft). Note that measuring the kinetics of such data is highly problematic due to the fact that the curve shape near the onset and end of each event is distorted by the sinusoidal variation; hence, analysis of this is not presented here.

During electrophysiological recordings, alternating current sources in the vicinity of the recording equipment can introduce a 50–60 Hz “hum” to recorded data. Therefore, as a final and strict test of our software's event detection ability, we generated a mock recording in which a 0.2 nA, 50 Hz sine wave plus 0.1 nA correlated random noise were added to events with amplitudes in the range 0.5–2.5 nA, with decay constants in the range 5–25 ms (for comparison, the period of a 50 Hz sine wave is 20 ms). Despite the fact that the half-width of the events was of the same order of magnitude as the sinusoidal fluctuations, and the smallest event amplitudes were comparable to the maximum peak-to-peak range of the noise (0.6 nA), true events were still detected, while the amplitude peaks of the hum that did not coincide with events were ignored (Fig. 4*A,B*). In a recording containing 142 true events, SimplyFire detected all but one of them, while the commonly used software MiniAnalysis (Synaptosoft; see now defunct website: https://web.archive.org/web/20210614022620/http://synaptosoft.com) missed seven events. Furthermore, amplitudes were reported with comparable accuracy to MiniAnalysis using equivalent parameter values, provided that the parameters were configured to make the distance between the baseline averaging window and the peak approximately equal to the period of the hum (in our case, ∼20 ms).

**Figure 5.**
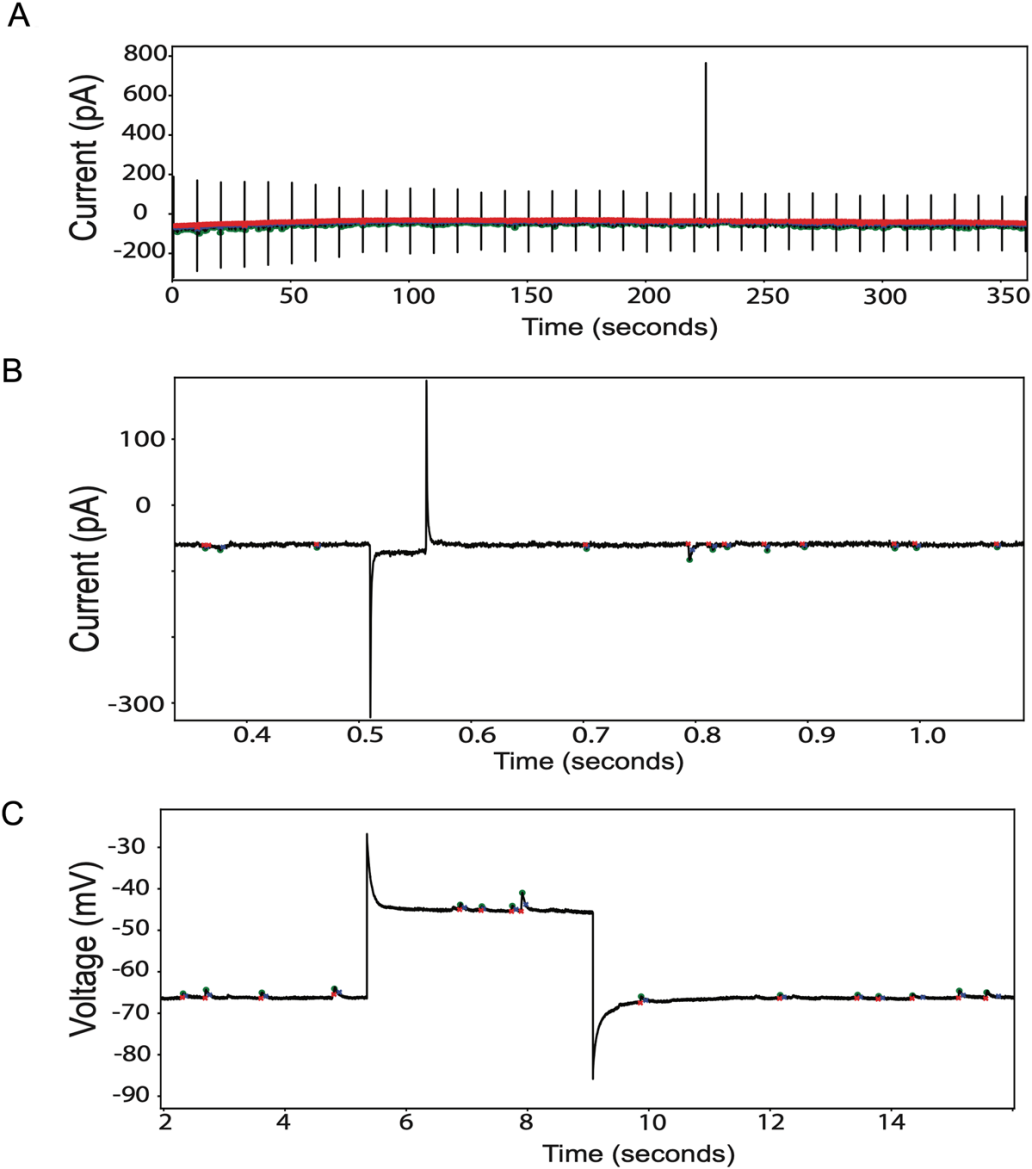
Testing with data containing high-amplitude artifacts. ***A***, Overview of a recording of mEPSPs from cultured human iPSC-derived glutamatergic neurons. Repeated artifacts correspond to a small current pulse to test changes in membrane input resistance. ***B***, A short window of the recording in (***A***), showing the detection of mEPSCs and rejection of the artifactual current spikes. ***C***, A short window of a recording of postsynaptic mEJPs from *Drosophila* larval NMJ, containing a single complex artifact consisting of two large voltage spikes at either end of a deviated baseline. The mEJPs are detected before, within, and after the artifact.

In testing our software, we also used several recordings containing high-amplitude artifacts, both from larval NMJ and from human iPSC-derived neurons in culture (Fig. 5*A,B*). When running SimplyFire on these recordings, we were able to configure the software to detect true events throughout while ignoring these artifacts, provided that the maximum amplitude cutoff chosen is between the maximum amplitude of the events and that of the artifacts (in the cases shown, a cutoff of ∼100 pA for mEPSCs in human neurons and ∼10 mV for mEJPs in larval NMJ). In any case, the presence of artifacts does not interfere with the detection of true events, even when, as seen in Figure 5*C*, events occur within a reasonably flat baseline that is nonetheless displaced considerably by an artifact from that in the remainder of the recording.

**Figure 6.**
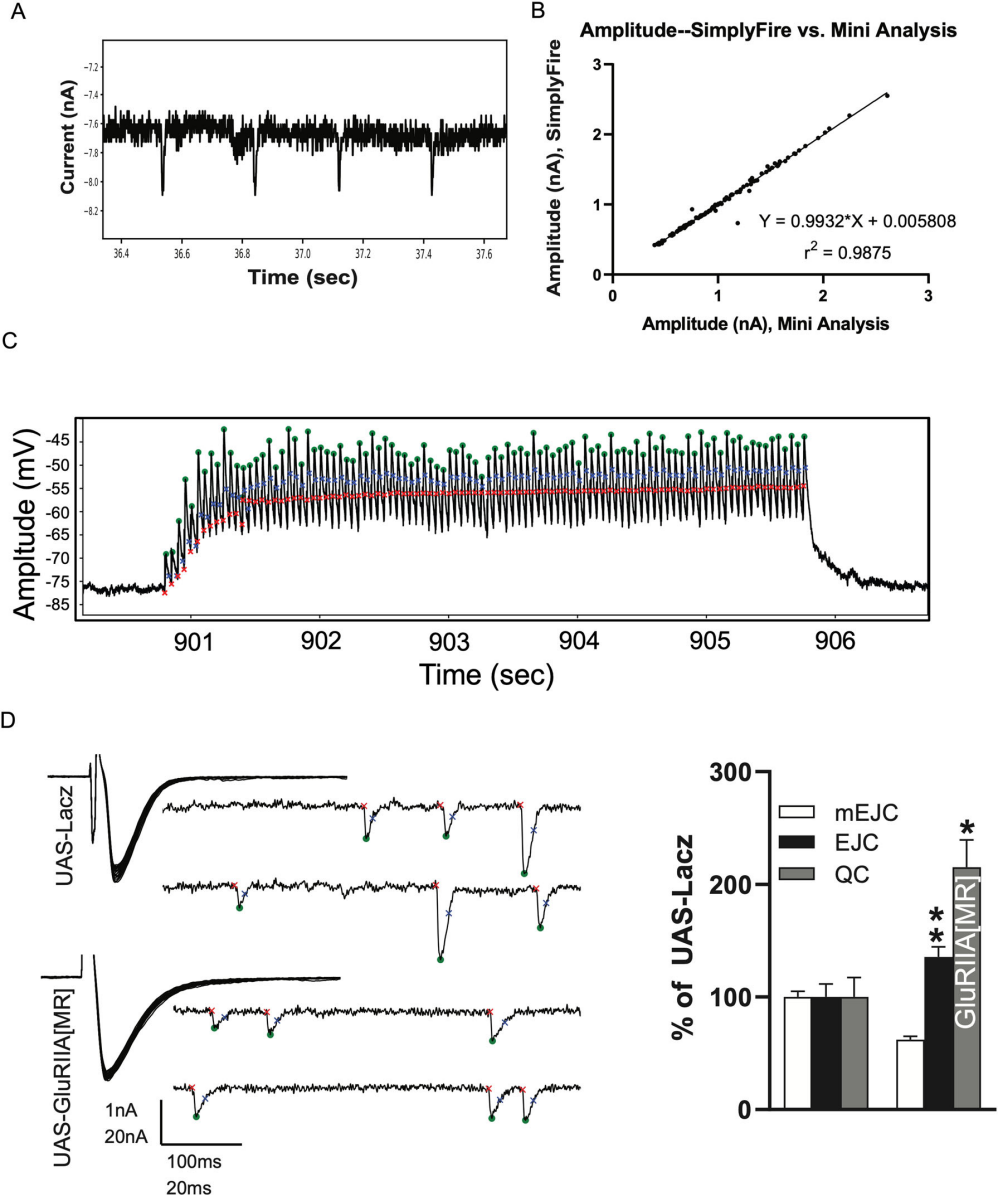
***A***, A sample of in-house data from the *Drosophila* larval NMJ used to compare SimplyFire with MiniAnalysis (Synaptosoft). ***B***, Comparison of detected amplitudes for the two software packages on data in (***A***). ***C***, Detection of individual evoked synaptic/junctional potentials (EJPs) at high-frequency (20 Hz) at the larval NMJ. Notice early facilitation that persists through the recording. This requires that the compound detection option is enabled (see text). ***D***, SimplyFire detects the difference in mEPSC amplitudes of GluRIIA mutants. Markers depict events that were detected by SimplyFire as a miniature synaptic event. The detected miniature postsynaptic currents in the wild type (left) are significantly larger in amplitude than the miniature events detected for the mutant (center), although the evoked currents (superimposed traces in the upper left of the panels) are of similar magnitude. The quantitation of the mEPSCs, EPSCs, and QC is shown in the right panel.

In order to compare the performance of SimplyFire with the commonly used MiniAnalysis software, we analyzed a sample of mEJC recording from larval NMJ. We found that the two programs highly agreed on the amplitudes of the detected minis (Fig. 6*A,B*). Finally, we tested whether the “compound” event detection feature, which is useful for detecting events that begin before the preceding event has decayed to baseline, can be used to analyze high-frequency trains of evoked synaptic potentials at the larval NMJ. As shown in Figure 6*C*, the individual events in such a series are detected, provided that the search window and the minimum separation between successive events are set short enough. The reader should be aware, however, that the amplitudes reported should be recalculated to present the amplitude from baseline measurements. The event detection follows the assumption, integrated into the Mini plugin, that overlapping events are summed, meaning that the amplitudes of later events are measured from the point to which the previous event had decayed rather than from the original baseline.

Spontaneous and evoked synaptic events are commonly analyzed to investigate mechanisms of synaptic homeostasis and synaptic compensation. At the *Drosophila* larval NMJ, genetic or pharmacological inhibition of postsynaptic glutamate receptor subunit GluRIIA reduced the average size of miniatures (mEPSCs/mEJCs), which triggers a retrograde enhancement in presynaptic neurotransmitter release also known as presynaptic homeostatic plasticity (PHP; Petersen et al., 1997; Frank et al., 2006; Penney et al., 2012). Figure 6*D* shows an example of PHP as a result of the postsynaptic expression of expression of GluRIIA[M/R], a dominant negative mutant (DiAntonio et al., 1999). In larvae expressing GluRIIA[M/R], mEPSC amplitudes are reduced while EJCs are maintained via presynaptic compensation. In order to demonstrate that the algorithm in SimplyFire can detect and quantify differences in spontaneous miniature synaptic event amplitudes, we compared the EJC, mEPSC, and quantal content (QC) of larvae expressing GluRIIA[M/R] to those expressing LacZ (control). As previously described, larvae expressing GluRIIA[M/R] showed >45% reduction in average mEJP amplitude and a compensatory increase in QC of >175% compared to control (Fig. 6*D*). The EPSC was not significantly different between the two genotypes.

## Discussion

We have created a freely available, open-source, and extensible software tool for the analysis of miniature postsynaptic events and for some other common tasks in electrophysiology data processing, such as noise filtering and baseline subtraction. It features a simple, intuitive user interface for interactive event detection and allows plugins to be created using the Python programming language to perform other tasks. This allows users of our software to distribute additional tools to the electrophysiology community.

We have verified the accuracy of our software on both synthetic data sets and synaptic recordings from *Drosophila* and shown it to be comparable to that of existing tools. The only cases where SimplyFire poorly fits data involve noisy events that are difficult for almost any software. In order to assist in dealing with such cases, a large range of detection parameters can be tuned and used to filter candidate events. This should extend the applicability of our software to a variety of systems where typical events differ greatly in amplitude and kinetics.

While other capabilities can be added by extending the software, the current plugins in SimplyFire can perform post-acquisition processing of the recording data, including noise reduction via filtering, baseline subtraction, and trace averaging. By utilizing such capabilities, SimplyFire can be incorporated into the experimental workflow immediately after data acquisition. We also added a plugin to analyze evoked synaptic activity amplitude, which can be performed by calculating the peak amplitude of the averaged trace. Details of each plugin can be found in the software documentation (https://simplyfire.readthedocs.io/).

The software is available as a stand-alone “zipped” executable or as a Python package. For Python users, SimplyFire can be installed using the “pip” package manager, while a “zipped” folder containing a Windows executable and all its dependencies is downloadable from the project's GitHub page (www.github.com/HaghighiLabBuck/simplyfire). The ready-to-run executable is the most accessible for users unfamiliar with the Python package ecosystem, whereas the package is useful for those who want to adapt and customize the software for their particular needs.

## Synthesis

Reviewing Editor: Niraj Desai, National Institute of Neurological Disorders and Stroke Decisions are customarily a result of the Reviewing Editor and the peer reviewers coming together and discussing their recommendations until a consensus is reached. When revisions are invited, a fact-based synthesis statement explaining their decision and outlining what is needed to prepare a revision will be listed below. The following reviewer(s) agreed to reveal their identity: Attila Szucs, Dion Dickman. Note: If this manuscript was transferred from JNeurosci and a decision was made to accept the manuscript without peer review, a brief statement to this effect will instead be what is listed below.

Two reviewers and I read your manuscript. We all found that SimplyFire has the potential to be quite useful to the neuroscience community. However, we all agree that the program should be tested more and that some problems (listed below) addressed. This likely requires additional work on the software.

REVIEWER 1

The manuscript entitled "SimplyFire, An Open-Source, Customizable Software Application for Analysis of Synaptic Events" by Mori et al. introduces a new software tool developed by the authors and designed for the analysis of spontaneous or miniature postsynaptic current recordings. While several chapters of the manuscripts read as a documentation for the software, the authors also demonstrate the operation of the program using synthetic and biological time series and describe the mathematical considerations behind the algorithms. As a general remark, I would recommend putting more empasis on the validation of the software using a variety of input time series, and experimental conditions (hum, drift, artifacts in the recordings). The analysis of synthetic data and the comparison of the performance of the program with that of the well-known Mini Analysis tool are surely nice, but I would suggest to show more examples and applications. As one interesting problem, how the program can cope with recordings that contain EPSCs and IPSCs with variable kinetics.

The work of Mori et al. is clearly important and they are addressing a need for fast, convenient and user-friendly solution for mini analysis. In the view of this reviewer, the majority of experimenters who record postsynaptic current data on a daily basis would happily employ a stable and easy-to-use analysis tool in their work instead on developing their own Python or Matlab scripts. Even commercially available solutions for mini analysis are ineffective, sometimes frustratingly slow and inflexible. Hence, a standalone program with compact installation package and ready to use for researchers who are not experts in program languages would be a valuable asset.

SymplyFire meets several of these requirements and it can certainly aid the work of the experimenters. It is certainly a However, there is still room for improvement regarding the robustness of the event detection algorithms and how users can interact with the program. The best way to get familiar with a new electrophysiological analysis software is to test it on various types of real-world recordings. The data I tested are from cortical neurons, contain pretty low amplitude minis and some slight drift in the recordings baseline. The program worked, in most cases, as I expected, the events were detected within 10 s and their extracted parameters were shown. Based on the analysis of several files, I found the following issues.

• The program failed to detect any mEPSCs when the recording contained one or more high amplitude artefacts. Typically, events were detected until the first large amplitude peak and nothing after that. Such artefacts can occur when there is a sudden electrostatic discharge in the vicinity of the recording device or perfusion system.

• One potential remedy for such errors would be the option to ignore artefacts. Automatic or manual artefact detection can be implemented and the program will ignore everything that falls within the artefact windows. Still, without artefact removal, the clean sections of the recording should be analyzed as expected.

• I am not sure if the method for calculating the baseline level preceding the event is the best. The program looks for local extrema, the baseline markers (red crosses) typically appear on local peaks in the signal. I understand that moving averaging is used here, but very fast jumps in the signal in th vicinity of the event onset can be a problem. The same argument can be made for the peak detection. Well, I understand that even the most trusted low pass filters can mislead the program to find the correct baseline level at the event onset, but it might be worth to explore additional methods and discuss their performance in the manuscript.

• Hum (50/60 Hz) in the recordings can result in false positives that I was able to identify visually. Certainly, this type of noise can fool many event detection algorithms, still, it would be nice to address this issue in the discussion of the manuscript and offer potential solutions to minimize the false detections.

• Pressing the 'Find in window' button sometimes suspends the program, looks like an infinite loop somewhere.

• Running the detection on the entire recording ('Find all') can also slow down after analyzing several ABF recordings. This is not very easy to predict, some files are quickly analyzed, others need minutes.

• Changing the X and Y-scales is a little bit awkward, the user needs to press the buttons with the magnifier symbol many times.

• The behavior of the Guide window is not stable. Traces disappear when pressing the 'Reanalyze' button. Sometimes the entire Guide window remains empty when clicking on a mini event in the Trace window (update problem).

• The motivation for the testing of the program with sinewave contaminated mock data is well justified (as shown in Supplementary figure 1), however, I would simulate more realistic scenarios here. The peak-to-peak fluctuations in typical patch clamp traces can be 5-10 pA (e.g. recording CA1 neurons in whole-cell conditions). I would suggest to construct a mock recording by creating a random white/pink noise trace with 10 pA fluctuations, then add a 50/60 Hz sinewave and then embed the events in this baseline. Small events should be also used for testing, i.e. those with similar amplitude as the noise.

REVIEWER 2

Mori et al. have developed a software analysis application, SimplyFire, that enables the quantification of electrophysiological events. This includes the standard analyses of miniature and evoked synaptic events, and a series of user-defined criteria to hone the analysis to the user's preferences and needs. It is an open source software free for researchers to use, and enables users to adapt new features using Python.

Overall this new software has the potential to become widely used in the field and an asset to researchers. The features and abilities of the current version of the SimplyFire software appear to be similar in interface and accuracy to that of Mini Analysis (Synaptosoft), which many in the field currently use (despite support being discontinued). It would benefit the manuscript to layout in what ways SimplyFire is superior to Mini Analysis, and/or develop additional features to help make the development of this software a clear advance. My expertise is not in coding or using Python, so I will limit my comments to strengthening the validation and utility of the software and suggestions to clarify some areas of the manuscript.

Suggestions:

1. Please comment in the manuscript on whether this program works equally well to quantify current clamp events (voltage; e.g. EPSP/mEPSPs) compared to voltage clamp (currents, EPSC/mEPSCs), which the manuscript highlights. Are rise and decay time constants calculated for each event similarly?

2. The interface and capabilities of SimplyFire appear quite similar to Mini Analysis, the free software that many in the field currently use. While the ability and flexibility for users to adapt and adjust SimplyFire for their own uses/needs using Python programing is a clear potential advantage, can the authors comment, or better yet, demonstrate, whether there is any advantage for researchers to use SimplyFire over Mini Analysis if they just need the standard analyses?

3. Can the "valley percentage" algorithm that the authors use to distinguish mini events that occur close to each other also be used to quantify EPSC amplitudes during high frequency trains of stimulation?
